# Degradome-focused RNA interference screens to identify proteases important for breast cancer cell growth

**DOI:** 10.3389/fonc.2022.960109

**Published:** 2022-10-12

**Authors:** Lena Hölzen, Kerstin Syré, Jan Mitschke, Tilman Brummer, Cornelius Miething, Thomas Reinheckel

**Affiliations:** ^1^ Institute of Molecular Medicine and Cell Research, Faculty of Medicine, University of Freiburg, Freiburg, Germany; ^2^ German Cancer Consortium (DKTK) Partner Site Freiburg, Freiburg, Germany; ^3^ German Cancer Research Center, Heidelberg, Germany; ^4^ Faculty of Biology, University of Freiburg, Freiburg, Germany; ^5^ Center for Translational Cell Research, Department of Internal Medicine I - Hematology, Oncology and Stem Cell Transplantation, Faculty of Medicine, University of Freiburg, Freiburg, Germany; ^6^ Center for Biological Signaling Studies BIOSS, University of Freiburg, Freiburg, Germany; ^7^ Comprehensive Cancer Center Freiburg (CCCF), University Medical Center, University of Freiburg, Freiburg, Germany

**Keywords:** breast cancer, protease, degradome, genetic screen, RNA interference

## Abstract

Proteases are known to promote or impair breast cancer progression and metastasis. However, while a small number of the 588 human and 672 murine protease genes have been extensively studied, others were neglected. For an unbiased functional analysis of all genome-encoded proteases, i.e., the degradome, in breast cancer cell growth, we applied an inducible RNA interference library for protease-focused genetic screens. Importantly, these functional screens were performed in two phenotypically different murine breast cancer cell lines, including one stem cell-like cell line that showed phenotypic plasticity under changed nutrient and oxygen availability. Our unbiased genetic screens identified 252 protease genes involved in breast cancer cell growth that were further restricted to 100 hits by a selection process. Many of those hits were supported by literature, but some proteases were novel in their functional link to breast cancer. Interestingly, we discovered that the environmental conditions influence the degree of breast cancer cell dependency on certain proteases. For example, breast cancer stem cell-like cells were less susceptible to depletion of several mitochondrial proteases in hypoxic conditions. From the 100 hits, nine proteases were functionally validated in murine breast cancer cell lines using individual knockdown constructs, highlighting the high reliability of our screens. Specifically, we focused on mitochondrial processing peptidase (MPP) subunits alpha (Pmpca) and beta (Pmpcb) and discovered that MPP depletion led to a disadvantage in cell growth, which was linked to mitochondrial dysfunction.

## 1 Introduction

Short hairpin RNA (shRNA) library-based RNA interference (RNAi) is a widely used method for large‐scale genetic loss of function screens, allowing for the unbiased discovery of cancer drivers, putative therapeutic targets, and genes with no previous links to cancer (1–3). shRNA libraries contain a heterogeneous mixture of different shRNA constructs targeting the whole genome (genome-wide screening) or a subset, i.e., all kinases, in so-called focused libraries. Although manifold genome-wide screens (4–6) and kinase-focused screens (7–9) were published in cancer cells in the last years, the study of an important class of enzymes, the proteases, was neglected so far.

Proteases are enzymes that irreversibly hydrolyze peptide bonds. Thereby, they (in)activate, degrade, or change the subcellular location of other proteins (10–12). Thus, they are essential for most physiological functions, and proteolytic activity is tightly controlled in cells, tissues, and body fluids. The complete set of proteases expressed in one organism or tissue is frequently defined as the degradome (13). According to the degradome database, 588 human and 672 murine protease and protease-like genes are currently known (13, 14). Degradome dysregulations are typical for cancers, facilitating or impairing tumor progression (10–12). Consequently, several proteases have been identified to be involved in all steps of cancer progression. Among the most studied groups of proteases are matrix metalloproteases (MMPs) that can promote tumor invasion by proteolysis of extracellular matrix and basement membrane components. In addition, they facilitate tumor growth by processing of bioactive molecules, e.g., growth factor receptors (15). Nevertheless, analysis of protease contribution to cancer has run into a bottleneck, with some proteases being extensively studied, e.g., MMPs or caspases (10–12), whereas the role of many degradome proteases remained understudied.

This paper presents a functional high-throughput degradome-focused RNAi screen to investigate the importance of all degradome-encoded proteases in murine breast cancer cell proliferation and/or survival. We chose to apply this screening method firstly to breast cancer cells because breast cancer is the most commonly diagnosed cancer in women and a global health burden with approximately 2.3 million new cases and 685,000 deaths in 2020 worldwide (16, 17). In 2022, 290,560 new cases and 43,780 deaths are estimated to occur in the United States alone (18). For our degradome-wide RNAi screens, we employed a customized shRNA library targeting the entire murine degradome (19). Specifically, we made use of a third-generation shRNA backbone, the enhanced microRNA (miR-E), generated by optimization of the native human miR-30a scaffold (20). Thus, miR-E constructs show increased pri-miRNA processing leading to higher mature shRNA levels that in turn boost potency and make miR-E constructs more useful for single-copy integration experiments (20, 21). Moreover, we employed an advanced RNAi vector system with inducible promoters for coexpression of the miR-E and a fluorescent reporter combined with a constitutive fluorescent marker to monitor vector integration. We have recently successfully applied this degradome-wide screening approach in a synthetic lethality setting (19). Now, we use it to define sets of proteases enabling or promoting breast cancer cell growth.

It is important to consider that tumors are complex tissues built from multiple cellular and genetically heterogenic cell subpopulations (22, 23). To reflect this, we chose to integrate the degradome-targeted library into two murine breast cancer cell lines with different properties, both previously generated by us from the transgenic MMTV-PyMT metastatic breast cancer model (24). The PyB6-313 cell line is an immortalized epithelial breast cancer cell line generated on a C57BL/6J mouse background (25). In contrast, PyMG-816 cells were immortalized from a primary CD24+CD90+CD45- tumor subpopulation from MMTV-PyMT mice with an FVB background and were shown to have cancer stem cell (CSC)-like properties (26). CSCs are a small tumor subpopulation that is of great importance for initiation, progression, and metastasis of cancer and is particularly problematic as the source of therapy-resisting cell populations (11, 27). It was previously shown that the PyMG-816 cells display a phenotypic and molecular plasticity when transferred from nutrient-deprived hypoxic (3% O_2_) stem cell conditions to nutrient-rich normoxic (21% O_2_) culture conditions (26). To prevent differentiation, the cells were normally kept in basement membrane extract (Cultrex™)-supplemented culture with only 3% oxygen content. Cells transferred to normoxic conditions increased in cytoplasm content and became polynucleated and more spindle shaped with close cell–cell contact (26). Functionally, normoxic culture conditions decreased anchorage-independent cell growth, impaired cell motility, and increased cell growth and metabolic activity *in vitro* as well as reduced lung metastasis formation in orthotopic transplantation models *in vivo*. Interestingly, transcriptome analysis revealed differently regulated protease messenger RNA (mRNA) expression upon changed environmental conditions (26).

By applying the miR-E library for our protease-focused genetic screening, we aim to investigate protease dependency in breast cancer cells and to discover proteases that have been overlooked in breast cancer so far. The use of two phenotypically different cell lines, one of them displaying CSC-like properties, reflects at least in part the tremendous cellular heterogeneity in tumors. In addition, the phenotypic plasticity of the PyMG-816 cells could be utilized to elucidate on the role for proteases under changed environmental conditions. We further employed inducible protease knockdown breast cancer cell lines to functionally validate our screens, whereby we, among other hits, identified and *in vitro* validated the mitochondrial processing peptidase (MPP) subunits alpha (Pmpca) and beta (Pmpcb) as important for breast cancer cell growth.

## 2 Results

### 2.1 Generation of murine degradome-targeted breast cancer cells for genetic screens

**Figure 1:**
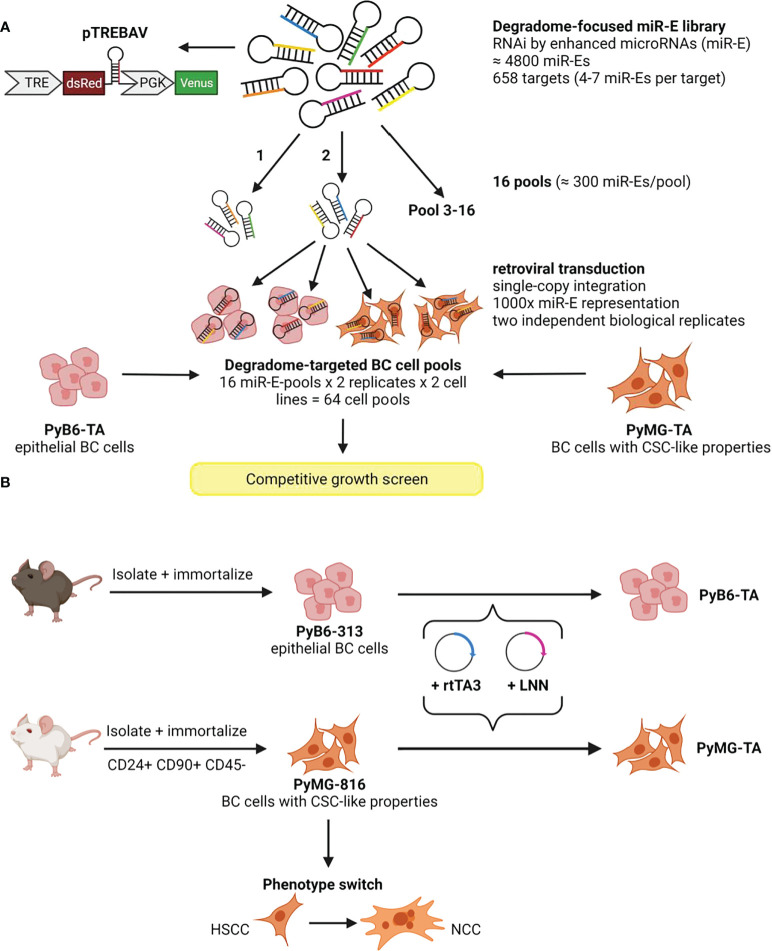
Generation of two degradome-targeted breast cancer cell lines. **(A)** Integration of a degradome-focused miR-E library into two MMTV-PyMT mouse model-derived breast cancer cell lines (PyB6-TA/PyMG-TA) leading to 64 cell pools for competitive growth screens. The miR-E library was inserted into the double-fluorescence Dox-inducible TRE-dsRed-miR-E-PGK-BSDr-2A-Venus (pTREBAV) vector; broad scheme; miR-E guide sequence in orange. TRE, tetracycline response element; PGK, phosphoglycerate kinase. **(B)** Establishment of MMTV-PyMT mouse model-derived breast cancer cell lines from different mouse backgrounds. BC, breast cancer; CSC, cancer stem cell-like; HSCC, hypoxic cancer stem cell conditions; LNN, luciferase; NCC, normoxic culture conditions; rtTA3, reverse tetracycline transactivator.

In our RNAi library, each protease transcript is targeted by 4–7 miR-Es (4,800 in total). The miR-Es are individually integrated into the doxycycline (Dox)-inducible double-fluorescence pTREBAV vector that enables time-controlled and traceable protease knockdown (**Figure 1A**). The pTREBAV vector constitutively expresses a Venus fluorescence reporter under control of the phosphoglycerate kinase (PGK) promoter. In addition, the vector carries an inducible dsRed reporter coupled to the miR-E sequence downstream of a tetracycline response element (TRE) that enables Dox-dependent gene silencing. The miR-E library was subdivided into 16 pools (300 miR-Es/pool) that were transduced into two self-generated breast cancer cell lines (PyB6-TA and PyMG-TA) expressing the reverse tetracycline transactivator 3 (rtTA3). Importantly, integration was performed under optimized conditions, ensuring single miR-E copy integration with 1,000× miR-E representation, i.e., 1,000 cells harboring the same miR-E construct. Library integration of the 16 miR-E pools was done in two independent biological replicates, leading to 32 degradome-targeted breast cancer cell pools for each cell line.

PyMG-TA and PyB6-TA cell lines were established from the parental PyMG-816 and PyB6-313 cells by integration of the rtTA3 as the second part of the Dox-inducible knockdown system, as well as the luciferase transgene (LNN; **Figure 1B**). The parental PyMG-816 and PyB6-313 cells have different properties and were both established in-house from the transgenic MMTV-PyMT metastatic breast cancer model (24). The CSC-like PyMG-TA cells were kept in basement membrane extract (Cultrex**™**)-supplemented culture with only 3% oxygen content to maintain their CSC properties, while changing to a normoxic growth medium induced a phenotypic switch (**Figure 1B**). Application of our degradome-targeted library to these biologically different cell lines allowed us to identify proteases generally important for breast cancer cell growth and proteases that are only important under certain environmental conditions.

### 2.2 Competitive growth screen performance is cell line-dependent

**Figure 2:**
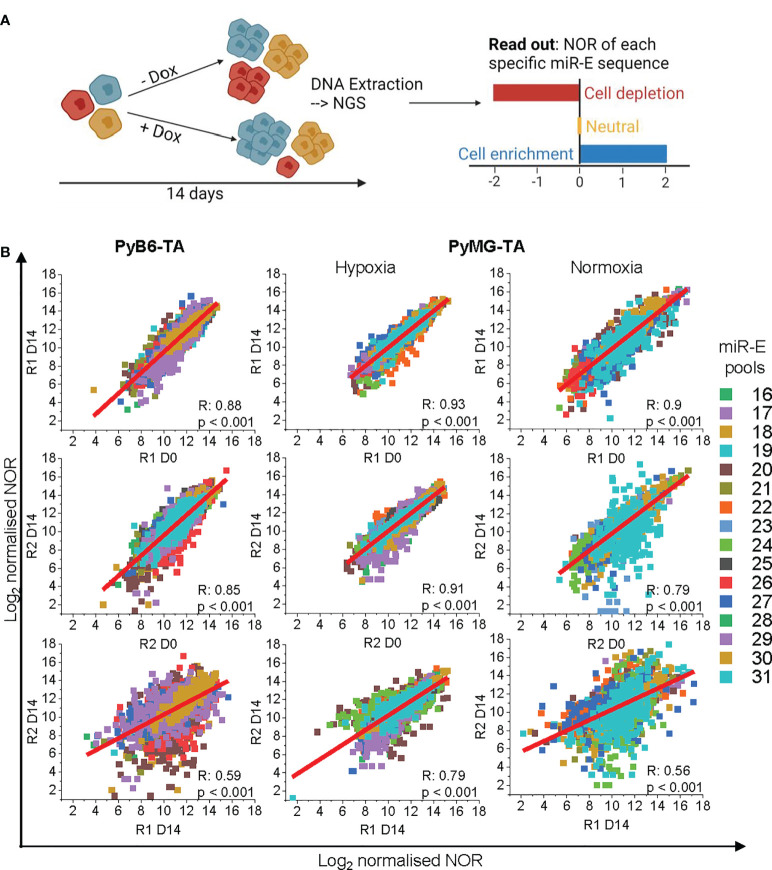
Competitive growth screen. **(A)** Schematic representation of the competitive growth screen setup. Cells carrying different miR-E constructs in different colors. NGS, next-generation sequencing; NOR, number of miR-E reads. **(B)** Correlation plots of Log_2_-transformed normalized sequencing reads of Replicate 1 (R1) and Replicate 2 (R2) compared between day 14 and day 0 within the replicated and day 14 between the replicates. miR-Es corresponding to specific pools in color code. For technical reasons, the 16 miR-E pools were termed pools 16–31. R, Pearson R correlation coefficient; p, Pearson R corresponding significance. OriginPro function: linear fit.

To investigate the protease dependencies of breast cancer cell proliferation and survival, we further applied our degradome-targeted cell pools to competitive growth screens (**Figure 2A**). Conceptually, in such screens, a loss of fitness due to impaired target gene expression leads to a dropout of the respective cells from the population, thereby identifying genes essential for cell survival or proliferation. Cells were cultured for 14 days in the absence or presence of Dox to induce protease silencing. At day 0 and day 14, DNA was extracted and used to PCR-amplify the miR-E cassettes followed by next-generation sequencing to compare the number of reads (NOR) for each miR-E insert between experimental conditions. Cells expressing miR-Es that downregulate proteases essential for cell growth become depleted in the Dox-treated cell population, leading to a lower NOR (cell depletion highlighted in red; **Figure 2A**). In contrast, targeting of proteases that negatively regulate cell growth results in overrepresentation of the respective cells upon protease knockdown (highlighted in blue; **Figure 2A**). However, targeting of most proteases was expected to have no effect (neutral; highlighted in orange; **Figure 2A**). Notably, the competitive growth screen in PyMG-TA cells was performed under two culture conditions, either under a nutrient-low 3% O_2_ hypoxic condition (hypoxia) or after transfer to a nutrient-rich normoxic culture condition (21% O_2_; normoxia). The two culture conditions were used to obtain insight into the role of proteases under different environmental settings and their involvement in the phenotypic plasticity of this cell line.

To assess data quality, the correlation of the normalized, bioinformatically trimmed, and log2-transformed NORs per miR-E was compared within replicates and between replicates (**Figure 2B**). The correlation of the NOR between day 0 and day 14 within each biological replicate was good (Pearson R ≥ 0.79), indicating no severe loss of specific miR-E abundance due to Dox-independent vector activation (leakiness) of the knockdown induction system. Correlation of the day 14 NORs between biological replicates was less (Pearson R = 0.56–0.79). However, because the biological replicates were generated via independent retroviral transductions with independently generated viral particles, this was not surprising.

**Figure 3:**
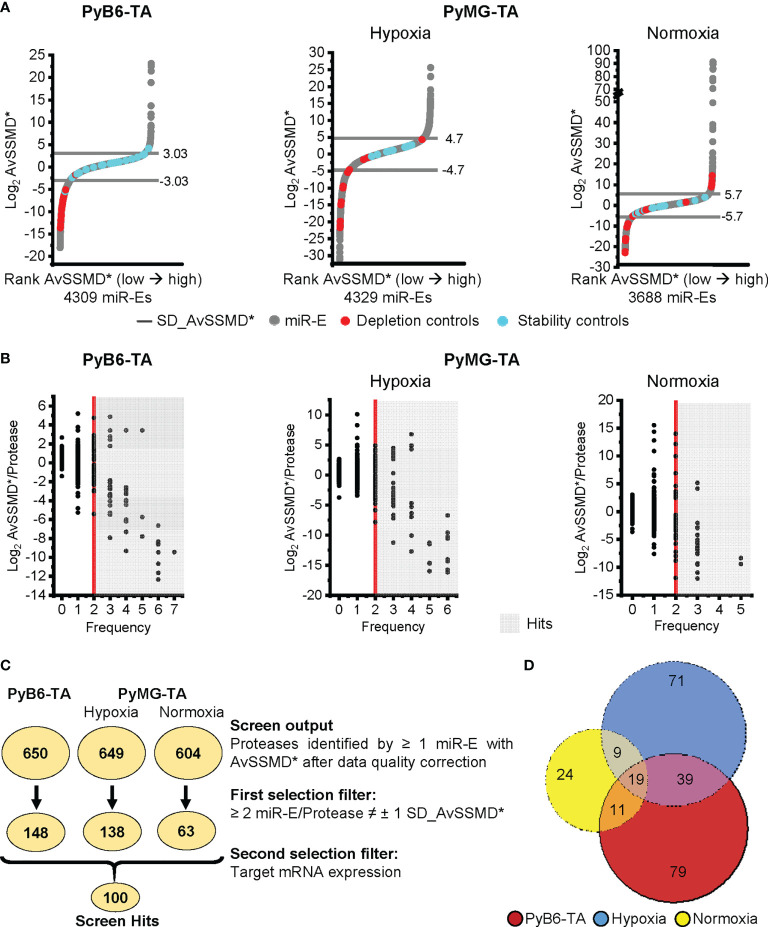
Competitive growth screen hit selection. **(A)** Distribution of Log_2_ AvSSMD* effect scores for all miR-Es in the competitive growth screens. Cell lines and conditions as indicated. Rank AvSSMD*: values ranked by size. Gray: Log_2_ AvSSMD* score of one miR-E. Blue: shRenilla/-Luciferase stability controls. Red: shRpa3-218/-Rpa3-457 depletion controls. Horizontal line: ± 1 SD_AvSSMD* of all miR-Es in the screen. **(B)** Dual flashlight plots. Black dot: Protease identified by minimum 1 corresponding miR-E with successfully calculated AvSSMD* after complete processing of sequencing reads. The term protease referred to all degradome-encoded proteins including proteases, protease-like proteins, and protease subunits. Hits highlighted in gray [≥2 miR-E/Protease outside ±1 SD_AvSSMD* (Frequency)]. **(C)** Summarized hit selection with number of identified proteases in circles according to the indicated criteria. ≠: outside. Searchable Excel file with the screen output and first selection filter hits in **Supplementary Tables S1** and **S2**. **(D)** Venn diagram of the hits from the first selection filter (252 proteases in total). Searchable Excel file with the input of the Venn diagram in **Supplementary Table S4**.

To analyze the functionality and robustness of the screen, the NOR was further used to assess effect size by calculating the robust strictly standardized median difference [AvSSMD* (28)]. The AvSSMD* represented the difference in miR-E abundance between day 14 protease-targeted (Dox-treated) and day 14 untreated cells compared to the background effect between day 14 untreated and day 0 samples. The validity of the screen was determined by controlling the distribution of the AvSSMD* scores for all miR-Es in the screen. As expected, the majority of miR-E constructs scored around zero because protease targeting did not significantly affect relative miR-E abundance and hence breast cancer cell growth (**Figure 3A**). One standard deviation (SD) from the AvSSMD* of all miR-E constructs in the screen (SD_AvSSMD*) was used to define the intrinsic variability of the screen, respectively, the effect region with no significant impact on breast cancer cell growth (SD_AvSSMD*: PyB6-TA ± 3.03 AvSSMD*; PyMG-TA hypoxia ± 4.7 AvSSMD*; PyMG-TA normoxia ± 5.7 AvSSMD*). Accordingly, miR-Es targeting proteases influencing breast cancer cell proliferation or survival would score as outliers outside **±**1 SD_AvSSMD*. To assess the functionality of the screen, two shRNAs targeting Renilla luciferase (shRenilla) or firefly luciferase (shLuciferase) transcripts, not present in mammalian cells, were incorporated into the library pools during transduction to serve as internal stability controls in the screens. Furthermore, two shRNAs targeting Rpa3 (shRpa3-457/-Rpa3-218) were incorporated. Because Rpa3 is essential for cell replication and proliferation (29), knockdown diminishes the respective cell clones in the population, making those constructs useful as depletion controls. In a stable system with only moderate intrinsic background variability, the shRenilla/-Luciferase stability controls are expected to score within the **±**1 SD_AvSSMD* intrinsic background variability. This expected distribution of the stability controls (blue dots; **Figure 3A**) was observed in all competitive growth screens in both cell lines. In contrast, the shRpa3-218/-Rpa3-457 depletion controls (red dots; **Figure 3A**) should deplete stronger than the intrinsic variance, leading to negative effect scores below -1 SD_AvSSMD*. Indeed, strong depletion of Rpa3 cell clones was observed for 28 of 29 depletion controls in PyB6-TA cells. Screen performance in PyMG-TA cells was less strong, especially under normoxic culture conditions, as 12 of 28 depletion controls scored below -1 AvSSMD* and some Rpa3 knockdown cell clones were even enriched in the Dox-treated population leading to positive AvSSMD* effect scores. This observation was most likely due to some leakiness of the pTREBAV vector in combination with a higher sensitivity of the CSC-like PyMG-TA cells toward Rpa3 knockdown. This resulted in a loss of shRpa3 cell clones in the reference sample, i.e., day 14 without Dox treatment. Nevertheless, the distribution of the internal controls demonstrated sufficient quality of the data and overall functionality of the screens for both cell lines, with better screen performed in PyB6-TA cells.

### 2.3 Competitive growth screens reveal the importance of many proteases in breast cancer cells

For hit selection, the Log_2_ AvSSMD* per protease was plotted against the number of miR-Es that scored outside **±**1 SD_AvSSMD* (Frequency; **Figure 3B**). Subsequently, we employed a preselection procedure that was previously applied by others (4, 30, 31). We only considered proteases for which at least two of the corresponding 4–7 miR-Es targeting this protease scored outside our **±**1 SD_AvSSMD* cutoff (Frequency ≥2; gray background; **Figure 3B**). In all three screens, most protease hits showed negative AvSSMD*/protease scores, indicating the importance of the particular protease for breast cancer cell growth. Notably, the competitive growth screen in PyMG-TA cells under normoxic conditions provided fewer hits with lower frequencies compared to the other screens.

**Figure 4:**
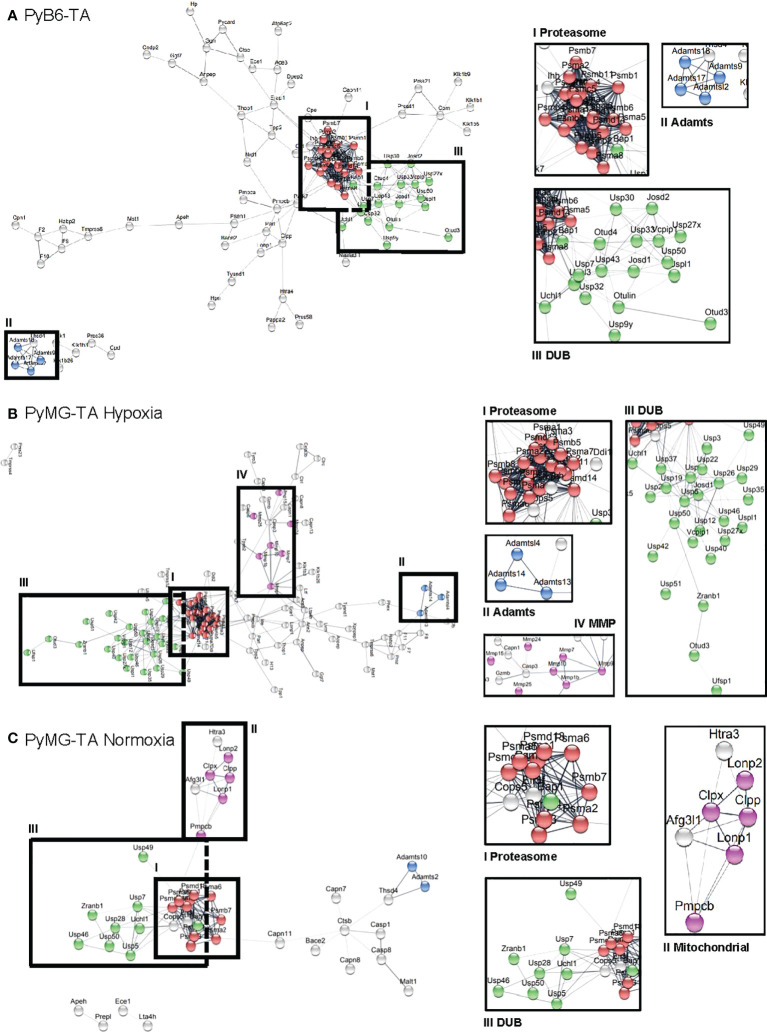
STRING-based cluster analysis. Analysis of hits from the competitive growth screens in PyB6-TA cells (**A**; 148 hits) and PyMG-TA cells cultured under hypoxic conditions (**B**; 138 hits) or normoxic conditions (**C**; 63 hits). Hits selected based on first selection criteria (≥2 miR-Es outside ±1 SD_AvSSMD*). Interesting clusters magnified on the right; associated proteins in color. Red: proteasomal subunits; Green: DUBs; Blue: Adamts; Pink: MMPs **(B)** or mitochondrial proteases **(C)**.

For the 658 protease genes initially targeted by the degradome-wide library, miR-Es corresponding to 650 proteases were identified in the competitive growth screen sequencing output in PyB6-TA cells (**Figure 3C**). In PyMG-TA cells, 649 proteases were identified upon cultivation under hypoxic conditions and 604 under normoxic conditions. Notably, the term protease referred to all degradome-encoded proteins including proteases but also catalytically inactive protease subunits and protease-like proteins (pseudoproteases). Applying the **±**1 SD_AvSSMD* cutoff that had to be reached by a minimum of two miR-Es targeting this protease as first selection filter, 148 hits were found in the PyB6-TA screen and 138 hits in the PyMG-TA screen in the hypoxic condition and 63 hits in the normoxic condition. In total, the hits from this first selection (**Figure 3C**) add up to 252 different proteases found in the three competitive growth screens (**Figure 3D**; **Supplementary Table S4**). Interestingly, 79 protease hits were only found in the screen in PyB6-TA cells and 71 in PyMG-TA cells cultivated under hypoxic conditions and 24 under normoxic conditions. Only 19 proteases were hits in all three cell lines. Search Tool for the Retrieval of Interacting Genes/Proteins (STRING)-based protein association network analysis of all 252 hits revealed a strong proteasome cluster in all screens composed of many proteasomal subunits (**Figure 4**). The proteasome cluster was accompanied by a cluster of deubiquitinases (DUBs). The importance of the ubiquitin-proteasome system (UPS) is well established in breast cancer cells (32–35). In PyB6-TA and PyMG-TA cells cultured under hypoxic conditions, an **“**a disintegrin and metalloproteinase with thrombospondin motifs**”** (Adamts) cluster was discovered that could not be found under normoxic conditions (**Figures 4A–C**). Furthermore, in PyMG-TA cells, an MMP cluster was found under hypoxic conditions and a cluster composed of different mitochondrial proteases was found under normoxic conditions (**Figures 4B, C**). These differences in overall protease clusters highlight the complex functions of proteases in different cell lines and culture conditions.

**Figure 5:**
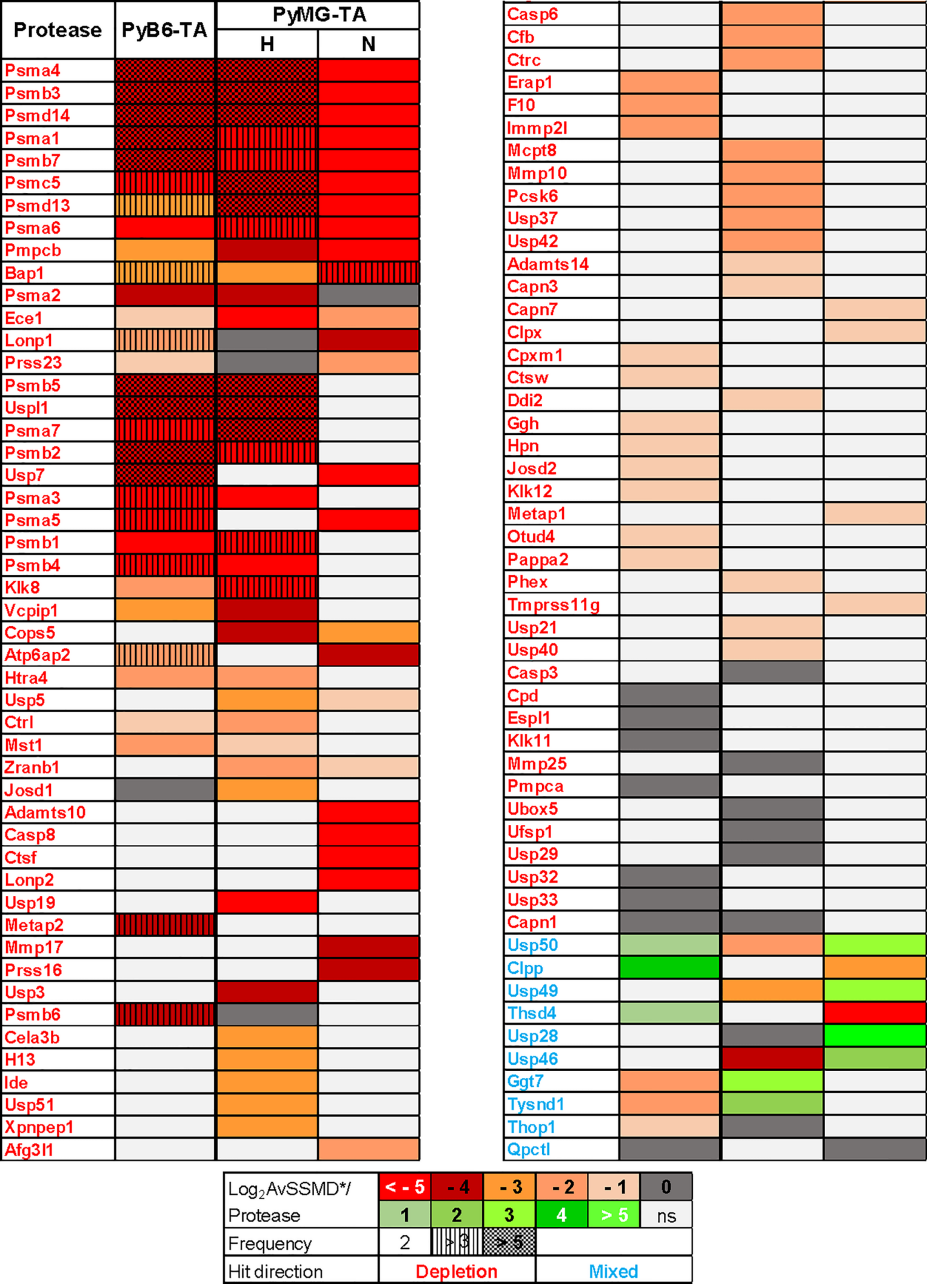
Competitive growth screen hits. Heat map of the competitive growth screen hits performed in miR-E library-transduced PyB6-TA or PyMG-TA cells cultured under 3% O_2_ hypoxic (H) or 21% O_2_ normoxic (N) culture conditions. Log_2_ AvSSMD*/protease as color code. Light gray (ns): not significant due to preselection criteria 1 (≥2 miR-Es/protease outside ±1 SD_AvSSMD*, filtered for sufficient target mRNA expression in both cell lines). Hit direction: red names (depletion); blue names (mixed). Frequency: number of miR-Es/protease outside ±1 SD_AvSSMD*. The term protease referred to all degradome-encoded proteins including proteases, protease-like proteins, and protease subunits.

Because low target mRNA expression levels could increase the chance for false-positive findings, hits were further filtered for sufficient miR-E-target mRNA expression (**Figure 3C**, second selection filter). Specifically, we excluded all proteases with mRNA expression below certain threshold levels, i.e., threshold PyB6-TA: FPKM 0.5 [data previously published (25); Gene Expression Omnibus (GEO) accession code GSE133328] and threshold PyMG-TA: arbitrary log2 expression level **<**6.2 [data previously published (26); GEO accession code GSE113826]. The remaining 100 screen hits were further depicted in a heat map to compare their effect strength (AvSSMD*/protease) and number of miR-Es targeting this protease outside the **±**1 SD_AvSSMD* (**Figure 5**). Notably, we excluded miR-Es showing positive AvSSMD*/protease scores (enrichment hits). Interestingly, 90 depleted protease hits (**Figure 5**; protease name indicated in red) were discovered with negative AvSSMD* scores per protease. Depletion hits represented proteases important for breast cancer cell proliferation or survival. As expected, many proteasomal α (Psma), β (Psmb), and γ (Psmc) subunits were among the top depletion hits. To address differences in protease dependencies between culture conditions or cell lines, we further searched for cell clones depleted in one cell line or environmental condition and enriched in the other. Those proteases, such as Usp46 and Usp50, were termed mixed hits (**Figure 5**; protease name indicated in blue).

In summary, the competitive growth screens performed in different cell lines and culture conditions yielded in a big data set of proteases involved in breast cancer cell growth. One hundred screen hits were identified by applying our strict selection criteria, thereby yielding the candidates for further analysis.

### 2.4 Functional validation of screen hits in breast cancer cells

To evaluate the hits from our competitive growth screens, we compared the 100 screen hits regarding their effect strength and robustness (number of miR-Es that showed the same effect) and subjected them to literature research. Nine hits were chosen as candidates, namely, cathepsin F (Ctsf), methionine aminopeptidases 1 and 2 (Metap1 and Metap2), matrix metalloprotease 17 (MMP17), MPP subunits alpha and beta (Pmpca/Pmpcb), 26S proteasome non-ATPase subunit 13 (Psmd13), and the ubiquitin-carboxyl terminal hydrolases 46 and 7 (Usp46 and Usp7). For those nine candidates, inducible single miR-E knockdown constructs were introduced into PyB6-TA and PyMG-TA breast cancer cells, each with two different miR-Es per protease. Those cells were then subjected to ***in vitro*** validation assays.

**Figure 6:**
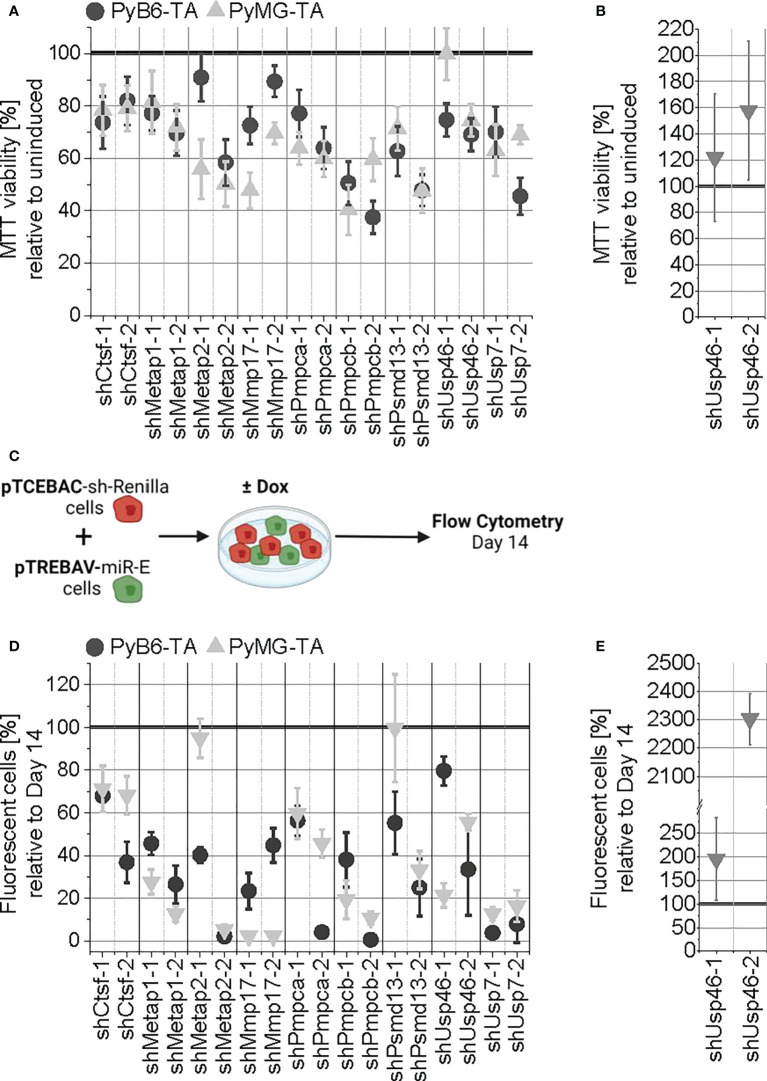
General validation of the competitive growth screen hits. **(A)** Short-term protease targeting in miR-E-transduced PyB6-TA and PyMG-TA cells. miR-E expression was induced for 5 days in total. Cell viability as mean ± SD (n ≥ 4) relative to untreated, uninduced cells. **(B)** Repetition of A performed under normoxic conditions for the mixed hit Usp46 in PyMG-TA cells (n = 5). **(C–E)** Long-term protease targeting in PyB6-TA and PyMG-TA cells. **(C)** Schematic experimental setup. **(D)** Flow cytometry-based percentage of Venus^+^dsRed^+^ fluorescent pTREBAV protease knockdown cells from living single cells at day 14 relative to unindicted Venus^+^ cells as average ± SD (n ≥ 3). **(E)** Long-term protease targeting assay performed under normoxic conditions for the mixed hit Usp46 in PyMG-TA cells (n = 5). For simplification, miR-Es are indicated by sh.

First, 3-(4,5-dimethylthiazol-2-yl)-2,5-diphenyltetrazolium bromide (MTT) assays were used to analyze the effect of short-term (5 days) protease targeting on cell viability. Targeting of all proteases impaired MTT viability to different extents, except for induction of shUsp46-1 in PyMG-TA cells (**Figure 6A**). Considering the two independent miR-Es for each target, the strongest effects with more than 45% MTT reduction were measured upon targeting of Metap2 in PyMG-TA and Pmpcb in PyB6-TA (≥50% MTT reduction) and PyMG-TA (≥40% MTT reduction) cells. To validate the difference in enrichment or depletion of Usp46-targeted PyMG-TA cells upon changed culture conditions in the competitive growth assay, the MTT assay was repeated for miR-E-Usp46-transduced PyMG-TA cells under normoxic conditions (**Figure 6B**). In contrast to hypoxic conditions, targeting of Usp46 increased MTT viability under normoxic conditions. This effect was most prominent upon induction of miR-E-Usp46-2, leading to 58% increase of cell viability compared to uninduced cells.

As MTT assays are rather short-term experiments, a competitive growth assay was performed to address the long-term effects of protease knockdown. For this, we utilized the double-fluorescence miR-E vector constructs incorporated into the cells. miR-E-transduced cells were cultured together with pTCEBAC-shRenilla-transduced control cells for 14 days in the presence or absence of Dox (**Figure 6C**). Because both vectors express different constitutive (pTREBAV: Venus; pTCEBAC: Cherry) and inducible fluorescences (pTREBAV: dsRed; pTCEBAC: Cyan), changes in relative abundance of the miR-E-transduced cells could be analyzed by flow cytometry. Relative to day 14, protease knockdown induction of any candidate, with the exception of Psmd13 by shPsmd13-1 in PyMG-TA cells, reduced the percentage of Venus^+^dsRed^+^ double-positive cells in the cell population (**Figure 6D**). Targeting Pmpcb (shPmpcb-2) or Usp7 nearly completely depleted the cells from the population. To validate Usp46 as a mixed hit from the screens, the competitive growth assay was repeated for miR-E-Usp46-transduced PyMG-TA cells under normoxic conditions (**Figure 6E**). Indeed, under normoxic conditions, Usp46-targeted cells were enriched in the cell population, most drastically upon usage of the second miR-E construct.

In summary, targeting of all nine protease candidates showed impairments in MTT viability and in competitive cell growth, therefore validating the overall quality of our initial competitive growth screens. In addition, Usp46 could be confirmed as a mixed hit with differential responses to its targeting in hypoxic and normoxic growth conditions. Because knockdown of Pmpcb showed the strongest growth-impairing effects in our murine breast cancer cells and as mitochondria are core organelles for cellular oxygen metabolism (36–38), we decided to further investigate the MPP.

### 2.5 The mitochondrial processing peptidase is essential for murine breast cancer cells

**Figure 7:**
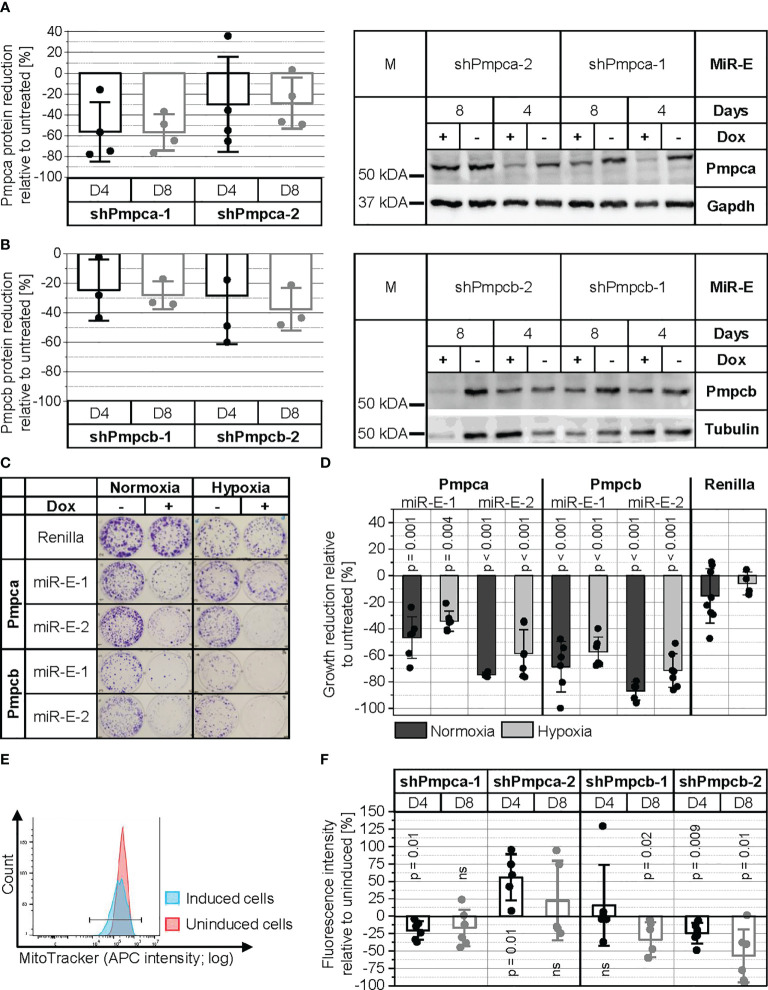
MPP is important for PyB6-TA cells. For simplification, miR-Es are indicated by sh. **(A, B)** Analysis of Pmpca **(A)** and Pmpcb **(B)** protein expression by Western blot in miR-E-transduced PyB6-TA cells cultured for 4 (D4) or 8 days (D8) ± Dox. Left: Quantification of Western blot data; mean reduction of protein level ± SD relative to uninduced cells normalized to tubulin or GAPDH. Right: Representative Western blots; tubulin or GAPDH as loading control; 25 µg protein loaded. M: marker. **(C, D)** Plate colony formation assay of miR-E-transduced PyB6-TA cells cultured ± Dox under normoxic conditions (21% O_2_, 8–9 days) or hypoxic conditions (3% O_2_, 12–14 days). ShRenilla-transduced cells as control. **(C)** Exemplary pictures. **(D)** Average cell growth reduction ± SD relative to untreated. Significance (p) one-sample t-test to 0 or two-sample t-test, equal variances assumed (n = 5–9). **(E, F)** Flow cytometry-based analysis of mitochondrial activity in miR-E-transduced PyB6-TA cells cultured for 4 or 8 days ± Dox by deep Red FM MitoTracker staining. **(E)** Exemplary median APC intensity. **(F)** Median APC fluorescence intensity ± SD relative to uninduced. Significance (p): one-sample t-test to 0 (n = 5–6). Ns, not significant.

Pmpca and Pmpcb are the two subunits of MPP, a protease complex essential for proteolytic removal of the mitochondrial import signal from the majority of mitochondrial proteins after their import into the mitochondrial matrix (36–38). First, the knockdown of Pmpca and Pmpcb in PyB6-TA cells was validated by Western blot (**Figures 7A, B**). A reduction of the Pmpca protein level by more than 50% was achieved by induction of the more potent miR-E-1 for 4 and 8 days. In the case of Pmpcb, between 20% and 40% reduction of protein levels was found after miR-E induction for 4 or 8 days. Although the protease knockdown efficiency was only moderate for both MPP subunits, cell growth was significantly impaired (**Figures 7C, D**). Pmpca knockdown in normoxic culture conditions significantly reduced colony growth by 47% and 75% for the two miR-E constructs, respectively. Targeting Pmpcb reduced colony growth even stronger, with 69% reduction upon induction of miR-E-Pmpcb-1 and 87% using miR-E-Pmpcb-2. In contrast, addition of Dox to shRenilla control cells did not significantly affect colony formation. Because MPP is known to be important for mitochondrial function (36–38), the colony formation assay was repeated under hypoxic conditions (3% O_2_) in which oxidative phosphorylation is limited. Cultivation under hypoxic conditions impaired general cell growth as visible from the weaker crystal violet staining independent of cell line and Dox addition (**Figure 7C**). Compared to hypoxia, the knockdown of Pmpca and Pmpcb impaired colony growth more prominently under normoxic conditions (**Figure 7D**). Due to the importance of MPP in mitochondrial function, we next validated if Pmpca or Pmpcb knockdown would compromise mitochondrial activity utilizing the MitoTracker**™** probe. This probe accumulates within active mitochondria and hence can be used to measure mitochondrial activity, in our case reflected by the median allophycocyanin (APC) fluorescence intensity (**Figures 7E, F**). As exemplary shown in **Figure 7E**, a left shift in APC intensity of the Dox-treated (blue peak) miR-E-Pmpca-1-transduced PyB6-TA cells compared to the untreated cells (red peak) indicated reduced mitochondrial activity upon Pmpca knockdown. Quantification of the MitoTracker**™** assays showed slightly reduced (≥17%) mitochondrial activity (fluorescence intensity) relative to uninduced cells upon targeting of Pmpca by miR-E-1 (**Figure 7F**). In contrast, induction of miR-E-Pmpca-2 significantly increased mitochondrial activity by 56% after 4 days, which was reduced to 22% after 8 days. Knockdown of Pmpcb significantly decreased mitochondrial activity upon usage of miR-E-Pmpcb-2 after 4 days (-34%) and both miR-E constructs after 8 days (-24%/-57%).

Taken together, knockdown of either MPP subunit led to impaired cell viability, colony growth, and mitochondrial activity. Thereby, effects were more prominent upon knockdown of Pmpcb.

## 3 Discussion

### 3.1 Degradome-focused RNA interference screens as a tool to investigate protease dependency of breast cancer cells

Although many proteases are linked to breast cancer (10–12), protease research has run into a bottleneck by only investigating already highly examined protease classes. Hence, the role of most of the 588 human and 672 murine protease and protease-like genes forming the degradome is still unknown (13, 14). In this work, we performed an RNAi-based genetic screen targeting the entire degradome in two murine breast cancer cell lines for an unbiased identification of proteases important for breast cancer cell proliferation and/or survival. Genetic screens are a widely used tool to efficiently discover cancer drivers, novel cancer-linked genes, and putative therapeutic targets (1, 2, 31), but genome-wide (4–6) or kinase-focused screens (7–9) missed out on proteases so far.

Our unbiased genetic competitive growth screens identified 252 protease genes involved in breast cancer cell survival/proliferation. STRING-based network analysis of first selection hits revealed that under all conditions, the biggest hit clusters comprised proteasomal subunits and DUBs (**Figure 4**), which are all components of the UPS (33, 34, 39). The UPS is central for protein turnover by proteasomal degradation of ubiquitin-tagged intracellular proteins controlling essential cellular functions such as cell death and proliferation. Because low expression of the miR-E target might increase the chance of off-target effects, we applied a second round of evaluations and filtered the hits obtained by the first selection filter for sufficient expression of the miR-E target mRNA, excluding all hits with low mRNA expression. By applying this multistep selection procedure, we detected 100 proteases important for breast cancer cell growth (**Figure 5**), of which many were supported by literature. Members of the UPS were among the strongest depletion hits. Indeed, the importance of a functioning UPS for cancer cells is well established (32–34), and proteasome inhibitors, which target the destructive part of the UPS, are useful anticancer drugs (40, 41). In all screens, the seven proteasomal α subunits (Psma) and at least six of the seven β subunits (Psmb) of the catalytic 20S core particle of the proteasome (42) were found as strong depletion hits (**Figure 5**), together with Psmc5, as well as Psmd13 and Psmd14 located in the 19S regulatory cap (42). In addition, many DUBs, such as Bap1 and Usp7, were found to be strong depletion hits in our screen (**Figure 5**). DUBs are part of the UPS and modify, trim, or remove ubiquitin chains on target proteins (43, 44). Thereby, DUBs change the **“**ubiquitin code**”** (number and position of attached ubiquitin molecules) that decides the fate of the tagged protein (45). Many DUBs are linked to cancer (34, 35, 46). For example, Usp7 is known to act as tumor-promoting in various cancer entities (47–50) including breast cancer (51–53). Together with the known cancer-promoting function of the UPS, the detection of many proteasomal components and DUBs as strong depletion hits proves the validity of our screening approach. In addition, many other depletion hits were supported by literature, e.g., Metap1 and Metap2. Metap2 is known to act as tumor-promoting in different cancers (54, 55) including breast cancer (19, 56). Metap1 is known to act as tumor-promoting in cervical cancer, fibrosarcoma, and lung cancer (57, 58), and we recently discovered a role for Metap1 in promoting the sensitivity of breast cancer cells to phosphoinositide 3-kinase (PI3K) inhibition (19). Also, MMP17 was already likened to breast cancer, promoting tumor growth (59). In line with our screen data, Usp7, Metap1, Metap2, and MMP17 could be validated as depletion hits *in vitro*, whereby targeting reduced MTT viability and competitive breast cancer cell growth (**Figure 6**).

Interestingly, although many of our depletion hits are known to promote cancer in general, our screen discovered that some of them were functionally connected to breast cancer. Among them was Lon protease 1 (Lonp1), being important in cervical cancer and colon cancer cells (60, 61), as discussed further below. Psmd14, one of our main depletion hits (**Figure 5**), stabilizes human epidermal growth factor receptor 2 (HER2) (62) and is upregulated in lung carcinoma associated with poor prognosis (63). Another protease was Uspl1 (ubiquitin-specific peptidase-like 1), a strong depletion hit in PyB6-TA cells and PyMG-TA cells cultured under hypoxia (**Figure 5**). In line with our data, Uspl1 mRNA expression was found to be upregulated in gastric cancer (64) and oral squamous cell carcinoma (65). In addition, genetic Uspl1 variants affecting its expression have been linked to breast cancer risk and cancer grade, indicating its importance in breast cancer (66). However, a functional link of those proteases to breast cancer biology has, until now, never been made. Other depletion hits, such as Psmd13 and the “peroxisomal matrix protein trypsin domain-containing 1” (Tysnd1), have to our knowledge not been postulated to be important for cancer growth. These proteases would be interesting to further investigate in the context of breast cancer. Here, we validated the role of Psmd13 for breast cancer cell growth *in vitro* by showing reduced MTT viability and colony growth upon targeting (**Figure 6**).

Because so many of our screen hits were supported by literature and all nine hits (Ctsf, Metap1, Metap2, MMP17, Pmpca, Pmpcb, Psmd13, Usp46, and Usp7) that we chose to validate individually showed impaired MTT viability and competitive growth in PyB6-TA and PyMG-TA breast cancer cells (**Figure 6**), we are confident that we established a robust and successful screen. More importantly, we were also able to detect proteases not linked to breast cancer before. Those could be interesting starting points for further investigation.

### 3.2 Breast cancer cells rely on mitocondrial processing peptidase for cell growth

Besides proteases corresponding to the UPS, the MPP subunit beta (Pmpcb) was one of the strongest depletion hits in both breast cancer cell lines (**Figure 5**). MPP consists of two subunits (α-MPP/Pmpca and β-MPP/Pmpcb), whereby Pmpcb has a catalytic function and Pmpca is involved in substrate binding and presenting and/or release of the cleaved peptide (36, 38, 67, 68). MPP is essential for the maturation of the majority of imported mitochondrial precursor proteins by proteolytic removal of their mitochondrial targeting sequence. Because mitochondrial activity is especially important in the energy-demanding brain, MPP dysfunction is associated with neurodegenerative diseases such as Friedreich ataxia (69), autosomal recessive cerebellar ataxia syndrome (70), neurodegeneration in early childhood, and cerebellar atrophy (71). The role of MPP in cancer, however, is rarely studied. In hepatocellular carcinoma (HCC) cells, Pmpcb knockdown led to apoptosis and tumor growth suppression by reactive oxygen species (ROS) formation and in turn suppressed Wnt/b-catenin signaling (4). Furthermore, Pmpcb silencing was shown to increase the susceptibility of murine and human HCC cell lines and HCC tumors to the multikinase inhibitor sorafenib (72). This combination decreased liver tumor burden and improved survival of the mice. In line with these HCC data, knockdown of Pmpca and especially Pmpcb in our PyB6-TA breast cancer cell line caused remarkable cell growth disadvantages (**Figures 7C, D**). Furthermore, knockdown of both protease subunits impaired MTT viability and competitive growth in PyB6-TA and PyMG-TA breast cancer cells (**Figure 6**). The antigrowth effect upon interference with MPP might be due to general impairment of mitochondrial function, as reduced mitochondrial activity was observed in PyB6-TA breast cancer cells upon knockdown of Pmpcb (**Figure 7F**). In general, mitochondrial function is known to support carcinogenesis *via* manifold processes, including oxidative phosphorylation to generate ATP, generation of ROS, and synthesis of precursors for biomass accumulation (38, 73). Furthermore, the association of MPP to mitophagy/apoptosis *via* the phosphatase and tensin homolog induced kinase (PINK)1-Parkin signaling pathway might contribute to the importance of Pmpca and Pmpcb for breast cancer growth, as already shown in human breast cancer MDA-MB-231 and MCF7 cells (74, 75). In those cells, inhibition of human Pmpcb led to accumulation of full-length PINK [in healthy mitochondria cleaved by MPP (76)] leading to PINK1-Parkin interaction-induced mitophagy (74) and apoptosis (75).

Interestingly, we observed that Pmpcb or Pmpca knockdown-dependent growth reduction was approximately 20% stronger under normoxic conditions as compared to that under hypoxia (**Figures 7C, D**). We hypothesize that cells under hypoxic conditions are less dependent on mitochondrial metabolism. Indeed, cancer cells have been shown to adapt their energy production to environmental changes by switching between glycolysis and oxidative phosphorylation as main energy source (77). Actually, most cancer cells prefer aerobic glycolysis over oxidative phosphorylation to produce ATP, a phenomenon termed “Warburg effect” (78). In line with the observed stronger growth reduction upon interference with MPP in normoxic conditions (**Figures 7C, D**), it was shown in yeast that growth arrest induced by MPP deficiency was attenuated in respiration-deficient mutants that do not rely on mitochondrial metabolism (79).

### 3.3 Environmental conditions influence breast cancer cell dependencies for specific mitochondrial proteases

Our STRING-based network analyses of all hits from the competitive growth screens also showed differences in protease dependencies upon changed environmental conditions (**Figure 4**). In PyMG-TA cells cultured under normoxic nutrient-rich conditions, a cluster composed of different mitochondrial proteases was discovered. This cluster was absent under nutrient-low hypoxic culture conditions. Although some of the cluster proteases, such as Pmpcb and Lonp1, were depletion hits in all conditions, only PyMG-TA cells in normoxic culture conditions were dependent on Lonp2, Clpp, and Clpx for proper survival and cell proliferation. As explained for Pmpcb and Pmpca above, we first hypothesized that cancer cells are less dependent on mitochondrial energy production under hypoxic conditions due to their preference for glycolysis (77). This could explain why the knockdown of certain proteases, such as Lonp2, Clpx and Clpp, only affects breast cancer growth under normoxic culture conditions strong enough to be detected as a screen deletion hit. In addition, we hypothesize that the condition-specific appearance of a protease as a depletion hit is based on its function. As already pointed out above, MPP has general relevance for mitochondrial function (36, 38, 67, 68), which explains why its catalytic subunit Pmpcb scored as a depletion hit in all screens (**Figure 5**). Another mitochondrial protease found to be important for cell growth under all tested conditions was Lonp1. Lonp1 is an ATP-dependent mitochondrial protease that degrades imported mitochondrial matrix proteins to maintain cellular homeostasis (80). Indeed, Lonp1 upregulation was found in several tumors, including lung, cervical, and oral cancer, associated with a worsened prognosis (38, 60, 81). In line with our data on breast cancer cells, Lonp1 downregulation suppressed cervical cancer cell proliferation and induced cell death in colon cancer cells (60, 61). In contrast to Pmpcb and Lonp1, the “caseinolytic mitochondrial matrix peptidase proteolytic subunit” (Clpp) and the “ATP-dependent Clp protease ATP-binding subunit clpX-like” (Clpx) were only found to be depletion hits under normoxic conditions. Clpp and Clpx are both components of ClpXP, a protease that catalyzes degradation of misfolded and specifically tagged proteins in mitochondria, which is important to maintain oxidative phosphorylation (82, 83). The restricted function of ClpXP for oxidative phosphorylation might explain why its two components (Clpp and Clpx) were more important under oxygen-rich (normoxic) conditions. Clpp inhibition is known to result in respiratory chain dysfunction in acute myeloid leukemia (AML) cells (84). However, the impact of Clpp in cancer is complex. Clpp is overexpressed in AML, and Clpp inhibition decreases cell viability (84). Surprisingly, Clpp hyperactivation also induces cell death in leukemia and lymphoma cells (85). It appears that a well-balanced selective proteolysis of mitochondrial protein subsets is important for proper organelle function. Our data now propose a dependency of some breast cancer cell lines on Clpp and Clpx under certain environmental conditions (**Figures 4**, **5**).

To sum up, our data reveal that breast cancer cells are dependent on MPP, and knockdown of its subunits, Pmpca or Pmpcb, led to reduced cell growth likely caused by mitochondrial dysfunction. Interestingly, we found that environmental conditions influence the degree of dependency on protease function, making breast cancer cells less susceptible to depletion of mitochondrial proteases under hypoxic conditions.

## 4 Conclusions

Based on internal controls and validation of many screen hits by experiment and literature, we prove degradome-focused RNAi-based pooled competitive growth screens as a suitable discovery pipeline to analyze the role of proteases in breast cancer cell proliferation/survival. The usability of our screening approach has previously been validated in context of synthetic lethality screens (19). Applying multistep selection criteria, our screens yielded 100 proteases that were identified to be important for breast cancer growth. Many of those hits were supported by literature, whereby some hits were so far overlooked in the context of breast cancer (e.g., Lonp1, Uspl1) or cancer in general (e.g., Psmd13, Tysnd1). Furthermore, our screen revealed cell line-specific and environmental condition-based dependencies of breast cancer cells to proteases, especially for mitochondrial proteases.

In conclusion, our data provide novel insights into the dependencies of different breast cancer cells onto protease function and discovered environmental condition-dependent protease importance.

## 5 Methods

### 5.1 Plasmids

Dox-inducible double-fluorescence retroviral pTREBAV (TRE-dsRed-miR-E-PGK-BSDr-2A-Venus) and pTCEBAC (TRE-Cyan-miR-E-PGK-BSDr-2A-Cherry) vectors, previously described (19), were designed based on the pTRMPV vector (86), which was kindly provided by Dr. Scott W. Lowe (Sloan Kettering Institute, Memorial Sloan Kettering Cancer Center, New York, USA). They allow for Dox-inducible TRE-driven dsRed (pTREBAV) or Cyan (pTCEBAC) fluorescent protein expression coupled to the miR-E expression. A constitutive PGK promoter drives Blasticidin resistance and Venus (pTREBAV) or Cherry (pTCEBAC) fluorescent reporter protein expression. The retroviral pMSCV-rtTA3-PGK-Puro and lentiviral pLNN plasmids [previously described (19)] were used to integrate the rtTA3 and LNN transgenes, respectively. Those vectors were kindly provided by Dr. Scott W. Lowe (Sloan Kettering Institute, Memorial Sloan Kettering Cancer Center, New York, USA) and Prof. Dr. Robert Zeiser (Department of Internal Medicine I, University Clinic, Freiburg, Germany), respectively. Single-target knockdown pTREBAV plasmids were generated as previously described (19) based on 97 nt oligonucleotides (97-mers) ordered from Sigma-Aldrich or Thermo (list in **Supplementary Table S3**) that encoded the specific shRNAs in a miR-E backbone.

### 5.2 Cell lines and cell culture

PyB6-TA and PyMG-TA cells (carrying the rtTA3 and LNN transgenes) were generated as previously described (19) *via* integration of the pMSCV-rtTA3-PGK-Puro and p-LNN vectors. PyB6-TA cells were cultured in Dulbecco's modified Eagle's medium (DMEM) high glucose, pyruvate supplemented with 10% fetal bovine serum, 1% penicillin/streptomycin, and 1% L-glutamine at 37°C with 5% CO_2_ and 21% O_2_. PyMG-TA stem cell-like murine breast cancer cells were cultivated in specific mammary stem cell medium under low-oxygen atmosphere [hypoxia (3% O_2_, 5% CO_2_, 92% N_2_)] at 37°C, as previously described (26).

miR-E-transduced PyB6-TA or PyMG-TA single-knockdown cell lines were generated as previously described (19) by retroviral integration of single-knockdown pTREBAV plasmids. Cell lines were tested for *Mycoplasma* contaminations prior to transduction and freezing in-house.

### 5.3 Degradome-targeted cells

For the degradome-wide knockdown, a customized degradome-focused miR-E library was used that was previously described (19). The miR-E library targeted 658 murine protease and protease-like transcripts with ≈4,800 miR-Es (4–7 miR-Es/target) incorporated into pTREBAV plasmids. The library was subdivided into 16 miR-E pools (≈300 miR-Es/pool).

Degradome-targeted PyB6-TA and PyMG-TA cells were generated as previously described (19) using specific protocols to ensure 1,000-fold miR-E representation and single-miR-E-copy integration. Briefly, PyB6-TA cells were cell cycle-synchronized prior to seeding by cultivation in starvation medium for 24 h [full DMEM 1% fetal calf serum (FCS)]. Infection of 13 plates per miR-E pool (0.75 × 10^6^ cells/10-cm plate) with ectopically packed miR-E library pool plasmid retrovirus (1:6 diluted in medium) supplemented with Polybrene (8 μg/ml) was done 16 h after seeding to infect shortly before the M-phase. Degradome-targeted PyMG-TA cells were generated due to the same protocol, but infection of 10 plates per miRE pool (0.75 × 10^6^ cells/10-cm plate) was done the next morning or 2 h after cell seeding. Blasticidin selection (10 µg/ml) started 48–72 h after infection, and transduction efficiency was controlled by flow cytometry. Blasticidin selection was continued until over 80% of fluorescent cells were obtained, repeating puromycin (4 µg/ml) and neomycin (500 µg/ml) selection to ensure the presence of the rtTA3 and LNN transgenes. Independently generated viral supernatant was used to perform two independent transductions per miR-E pool. Four miR30A backbone-based controls {stability controls: pTCEBAC-shRenilla (87); pTCEBAC-shLuciferase (88); depletion controls: pTREBAV-shRpa3-218 [termed Rpa3.276 in David et al. (87)]; pTREBAV-shRpa3-457 [termed Rpa3.455 in McJunkin et al. (88)]} served as internal quality controls and were incorporated during ecotropic virus production as one construct per miR-E pool (sequence information in **Supplementary Table S3**).

### 5.4 Virus production

Ecotropic virus was produced as previously described (19).

### 5.5 Competitive growth screen

Degradome-targeted PyB6-TA or PyMG-TA cell pools were cultured in their respective medium for 14 days ± Dox treatment (2 µg/ml). One miR-E pool was cultured per 15-cm plate (PyB6-TA) or 10-cm plate (PyMG-TA). Medium and Dox were changed every 2–3 days, cells were passaged regularly keeping 1.1 × 10^6^ cells/plate to maintain miR-E representation. In PyMG-TA cells, the screen under hypoxic conditions was performed as described above. For the normoxia screen, PyMG-TA cells were transferred to normoxic culture conditions 10 days prior to the start of the screen as follows. Cells were detached, centrifuged, resuspended, and seeded in full DMEM. Cells were further cultivated at 37°C with 5% CO_2_ and 21% O_2_ in full DMEM, maintained throughout the screen.

At day 0 and after 14 days of cultivation, genomic DNA (2 × 10^6^ cells/plate) was isolated using the Gentra Puregene Cell Kit (158767; Qiagen). Deep sequencing template libraries were generated by PCR amplification of the miR-E cassettes as previously described (19) resulting in PCR products (315 nt) that were tagged with standard Illumina P5/P7 adapters and a sample-specific 10-nt barcode sequence. PCR products were pooled to one sample for sequencing (theoretical sequencing depth of 1 × 10^6^ bp per initial sample) according to their relative abundance in gels to archive equal sequencing reads. The sequencing sample was further column-purified [QIAquick PCR purification kit (28104; Qiagen)], and the concentration was adjusted to 0.832 nM for optimal cluster generation. Next-generation sequencing was performed on a HiSeq4000 [German Cancer Research Center (DKFZ)/Genomics & Proteomics Core Facilities/TP3/High-Throughput Sequencing Unit] using 150-bp paired-end sequencing and the Illumina sequencing primer (5´-TAGCCCCTTGAAGTCCGAGGCAGTAGGCA). Alternatively, sequencing was performed on a HiSeq2500 (MPI for Immunology and Epigenetics Freiburg/Deep Sequencing Facility) or Nextseq500 (Faculty of Medicine Freiburg/Department for Pediatrics/Pediatric Genetics*)* utilizing 75-bp single-end sequencing, the Illumina sequencing primer for miR-E cassette readout, and a custom index primer (5´-CGCTCACTGTCAACAGCAATATAC) for sequencing the 10-bp barcode as index reads.

### 5.6 Analysis and selection of screen hits

Sequence processing was performed as previously described (19) including cleaning, pool-based normalization, Log_2_ transformation, and trimming [removal of extreme values or outliers; criteria see Hölzen et al. (19)] of the raw number of reads for each miR-E in the library per sample. R-scripts can be provided upon reasonable request to the first author. For calculation of effect strength, the robust strictly standardized median difference (AvSSMD*) calculated after a modified version of the method of moment estimate of the paired SSMD* by Zhang (28) was chosen, being suitable for screens with biological duplicates only. The AvSSMD was calculated as follows:

- FGR: NOR_Day14Dox/NOR_Day14

- BGR: NOR_Day0/NOR_Day14

Foreground (FGR): difference of a NOR of a specific miR-E between Dox-treated and untreated samples. Background (BGR): difference in NOR between day 0 and day 14 untreated samples. NOR: Number of reads of a specific miR-E in the respective sample. Day0: DNA isolated at day 0. Day14: DNA isolated at day 14 from untreated cells. Day14Dox: DNA isolated at day 14 from Dox-treated cells. If the corresponding Day14 sample was excluded due to bad quality during sample processing, the FGR was calculated as NOR_Day14Dox/NOR_Day0.

- SSMDR1* = (FGR_1-medianBGR)/MAD * √2

- SSMDR2* = (FGR_2-medianBGR)/MAD * √2

- AvSSMD* = Average (SSMDR1* + SSMDR2*)

- AvSSMD*/Protease = Average (AvSSMD*all miR-Es targeting the same transcript)

The SSMD* was calculated for each miR-E in both biological replicates independently [_1/_2 (Replicate 1 and 2)]. MedianBGR: Median of BGR from all miR-Es in both replicates. Median absolute deviation (MAD): 1.4826 * Median from the absolute values of BGRReplicate1-MedianBGR and BGRReplicate2-MedianBGR. AvSSMD* = Average of the SSMD* from both replicates, thereby allowing to keep the value of one replicate if the other is empty (use of only one is highlighting for score calculation).

Hits were defined as proteases with minimum two miR-Es per protease scoring outside of the ±1 SD from the AvSSMD*s of all constructs in the screen (SD_AvSSMD*). Following this, hits were filtered for miR-E target mRNA expression, selecting only hits with expression levels above defined thresholds {PyB6-TA: RNAseq FPKM >0.5; data previously published [GEO accession code GSE133328 (25);]; PyMG-TA: microarray arbitrary log2 expression level >6.2; data previously published [GEO accession code GSE113826 (26)]}.

### 5.7 MTT assay

Cells were cultured ± Dox (2 µg/ml) for 3 days prior to seeding onto 96-well plates [0.5 × 10^4^ cells/well (PyB6-TA); 0.8 × 10^4^ cells/well (PyMG-TA)] in triplicate. For the assay under normoxic culture conditions, miR-E-Usp46-transduced PyMG-TA cells were transferred to normoxic culture conditions 3–5 days prior to the start of the experiment, as described for the competitive growth screen, and were kept under these conditions throughput the assay. Cells were treated for 48 h ± Dox, following incubation for 1–6 h with indicator-free medium containing MTT (0.5 mg/ml). Plates were emptied, and Dimethyl sulfoxid (DMSO) was added to dissolve formazan crystals. The absorbance was measured at 570 nm (650 nm reference) using an EnSpire multimode plate reader. MTT viability was calculated by normalizing the 570-nm absorbance to reference readings at 650 nm. Following this, the MTT viability of Dox-treated samples was set relative to untreated cells averaged per triplicate. For calculation of means of biological replicates, values outside mean ±2 SD were excluded.

### 5.8 Long-term protease targeting flow cytometry assay

miR-E-transduced PyB6-TA or PyMG-TA cells were mixed with the respective pTCEBAC-shRenilla-transduced cells (≈70% to 30%), generating competitive growth conditions, and were seeded onto six-well plates [0.15 × 10^5^ cells/well (PyB6-TA)] or 24-well plates [0.4 × 10^5^ cells/well (PyMG-TA)]. Cells were cultured for 14 days ± Dox (2 μg/ml). For the assay under normoxic culture conditions, miR-E-Usp46-transduced PyMG-TA cells were transferred to normoxic culture conditions 3–5 days prior to the start of the experiment, as described for the competitive growth screen, and were kept under these conditions throughout the assay. Changes in relative abundance of pTREBAV-transduced cells were analyzed by flow cytometry comparing the constitutive and inducible fluorescence between Dox-treated and untreated samples at day 14. The percentage of Venus^+^dsRed^+^ double-positive cells from living single cells of Dox-treated day 14 samples was normalized to the percentage of Venus^+^ cells in the untreated sample. Following this, the mean percentage of fluorescent knockdown cells to uninduced cells was calculated from all biological replicates, whereby values outside mean ±2 SD were excluded.

### 5.9 Plate colony formation assay

Cells were separated (70–100-µM cell strainer) and seeded at single-cell conditions onto six-well plates. Plates were either further cultivated under normal culture conditions (normoxia; 21% O_2_) or were transferred to hypoxic culture conditions (3% O_2_). After 24 h, Dox treatment was performed (2 μg/ml). Medium and treatment were changed every 2 days. After 8–9 days (normoxia) or 12–14 days (hypoxia), cells were stained with 1% crystal violet in 20% methanol (10 min). Pictures were taken in raw format using a light desk and the Canon Powershot G6 camera. After converting the raw files to 800-dpi tiff files using Adobe Photoshop CS2, the ImageJ plugin Colony Area by Guzmán et al. (89) was used to calculate colony intensity percentage (further referred to as cell/colony growth). Reduction in cell growth was calculated relative to uninduced cells for each biological replicate as follows: Growth reduction [%] = (Intensity percent Dox/Intensity percent no Dox) - 100. For calculation of the mean growth reduction of biological replicates, values outside mean ±2 SD were excluded.

### 5.10 MitoTracker assay

The MitoTracker™ Deep Red FM Special Packaging Assay (M22426; Thermo) was used according to the provided protocol. Briefly, cells were cultured ± Dox (2 μg/ml) for 8 days, whereby the MitoTracker assay was performed at days 4 and 8. The day before the assay, cells (2 × 10^5^ cells/well) were seeded onto 24-well plates. The next day, medium was removed, cells were washed with Dulbecco's phosphate-buffered saline (DPBS), and 200 μl of the staining dilution [100 nM compound in DMSO diluted in FCS-free DMEM (1:5,000)] was added, following incubation for 30 min at 37°C. For flow cytometry, cells were transferred into a 96-well round-bottom plate, spun down (3 min, 280 rcf), and washed with fresh DMEM. Single living cells from untreated samples were gated for Venus^+^/APC^+^. Dox-treated samples were gated for Venus^+^/dsRed^+^/APC^+^. The change in median APC fluorescence intensities from Dox-treated Venus^+^/dsRed^+^/APC^+^ cells were normalized to untreated Venus^+^/APC^+^ cells, and the change in fluorescence intensity was calculated as follows: Fluorescence intensity [%] = (Venus^+^/dsRed^+^/APC^+^_Dox sample_ * 100/Venus^+^/APC^+^_no Dox sample_) – 100.

### 5.11 Flow cytometry

Cells were harvested, pelleted (5 min, 280 rcf), and resuspended in fluorescence-activated cell scanning (FACS) buffer (DPBS, 2% FCS, 5 mM EDTA). Following this, samples were separated (70–100-μm cell strainer) and transferred to FACS tubes or plates. Analysis was performed using the Cytoflex SFlow (Beckmann Coulter) and the FlowJo 7.6.5/10.6.0 software (BD Bioscience). Gated viable cells [forward-scattered area (FSC-A) vs. side-scattered area (SSC-A)] were further restricted to singlets [forward-scattered width (FSC-W) vs. height (FSC-H)]. Living single cells were further gated individually for different fluorescences depending on the experiment. All biological replicates were gated with the same defined gates.

### 5.12 Protein isolation and immunoblotting (western blot)

Cells were harvested by scraping on ice in phospho-Radioimmunoprecipitation assay (RIPA) lysis buffer [Tris-HCl (50 mM, pH 7.5), NaCl (150 mM), Triton X100 (1%), sodium deoxycholate (0.5%), sodium dodecyl sulfate (SDS) (0.1%), ethylenediaminetetraacetic acid (EDTA) (1 mM, pH 7), Natriumpyrophosphate (2.5 mM), β-glycerophosphate (1 mM), Natriumvanadat (1 mM), PhosStop Phosphatase-inhibitor mix (04906845001; Roche), Complete Ultra tablets (5892970001; Sigma-Aldrich) in ddH_2_O]. Cells were disrupted mechanically, and cell lysates (25 μg protein) were subjected to sodium dodecyl sulfate (SDS)-polyacrylamide gel electrophoresis (PAGE), following transfer to a nitrocellulose membrane (Hybond) by a wet blot system (BioRad). Membranes were blocked with 3% bovine serum albumin (BSA) in Tris-buffered saline (TBS)-Tween (0.1%, 1 h). Primary antibodies α-tubulin [T9026; Sigma (1:1,000)], Glyceraldehyde-3-phosphate dehydrogenase (GAPDH) [97166; Cell Signaling (1:1,000)], and Pmpca [sc-390471; Cell Signaling (1:5,000)] or Pmpcb [PA5-110185; Thermo Scientific (1:5,000)] were incubated overnight at room temperature. Membranes were washed with Tris-buffered saline with Tween20 (TBS-T) and incubated with the corresponding secondary goat-anti-mouse-horseradish peroxidase [A0168; Sigma (1:5,000)] or goat-anti-rabbit-horseradish peroxidase [111-035-003; Jackson Laboratories (1:5,000)] antibodies for 60–120 min at room temparature. Washing was repeated, and membranes were developed using the West Pico/Femto Chemiluminescent Substrate (34080/3 4095; Thermo Scientific). Chemiluminescent signal detection and analysis were done using the Fusion SL Detection System and FusionCapt Advance software (Vilber Lourmat). Protein quantification (volume under the signal peak) was done relative to α-tubulin or GAPDH (probed on the same membrane) employing the automatically set rolling ball function for background correction.

### 5.13 General statistical analysis and data presentation

Statistical analyses were carried out with OriginPro 2018/2020 (OriginLab). Quantitative data of independent biological replicates (n) were plotted as mean ± SD, if not stated differently. Technical replicates were corrected for SD ≥0.1. Values outside mean ±2 SD were excluded from calculating the mean of biological replicates. Statistical significance was determined by one-sample or two-sided two-sample t-test (p ≤ 0.05 significance level). General graphical depiction was done with OriginPro 2018/2020 (OriginLab) or BioRender.com. The R package “eulerr” was utilized to generate Venn diagrams (90) prior to the final optimization using Microsoft Power Point.

### 5.14 String analysis

STRING (91) analysis was performed on the competitive growth screen hits of the first selection criteria (≥2 miR-Es outside ±1 SD_AvSSMD*). Default analysis parameters allowing textmining, experiments, and databases as active interaction sources only were used, choosing confidence as meaning of network edges. STRING-based networks were further processed in Cytoscape 3.8.2 (92), removing unconnected nodes, changing node color and label size.

## Data availability statement

The original contributions presented in the study are included in the article/**Supplementary Material**. Further inquiries can be directed to the corresponding author.

## Author contributions

LH and TR designed the study and wrote the manuscript. LH conducted experiments and analyzed data. CM and JM designed the protease targeting miR-E shRNA library. CM developed the TREBAV vector, the shRNA library cloning protocol, the screening strategy and the protocol for miR-E deep sequencing. KS performed the experiments for **Figure 7**. JM performed sequencing library deconvolution and part of the bioinformatic sequencing data analysis. KS, JM, TB, and CM read and critically revised the manuscript. All authors approved the final version of the manuscript.

## Funding

The work was supported by the German Cancer Consortium DKTK (projects L627 and FR01-371) to TB, CM, and TR. This work was further supported by the Deutsche Forschungsgemeinschaft (DFG), under Germany’s Excellence Strategy (BIOSS-EXC-294), the Collaborative Research Centre 850 (projects B4 and B7), the Heisenberg-Professorship BR 3662/5 (to TB) and GRK 2606 (Project ID 423813989; to TR).

## Acknowledgments

The authors sincerely thank Pia Veratti (Department of Internal Medicine I, University Clinic Freiburg, Germany) for her expert technical assistance and cloning of the protease targeting miR-E shRNA library. We further thank Dr. Stephanie Ketterer and Dr. Larissa Hillebrand (Institute of Molecular Medicine and Cell Research, Faculty of Medicine, University of Freiburg, Germany) for providing the PyB6-313 and PyMG-816 cells. In addition, we thank Prof. Dr. Robert Zeiser (Department of Internal Medicine I, University Clinic Freiburg, Germany) for providing the pLNN vector and Dr. Scott W. Lowe (Sloan Kettering Institute, Memorial Sloan Kettering Cancer Center, New York, USA) for the pMSCV-rtTA3-PGK-Puro and pTCEBAC vectors. We thank Dr. Sophia Ehrenfeld (Department of Internal Medicine I, University Clinic Freiburg, Germany) for help with deep DNA sequencing. Furthermore, we thank Dr. Anett Ketscher and Prof. Dr. Christoph Peters (Institute of Molecular Medicine and Cell Research, Faculty of Medicine, University of Freiburg, Germany) for their valuable discussions. Finally, we thank the Genomics and Proteomics Core Facility of the German Cancer Research Center (DKFZ) in Heidelberg for their DNA-sequencing service, the Department for Pediatric Genetics at the Medial Center Freiburg, especially Tanja Velten, for access to their Illumina Nextseq500 DNA sequencing platform and the Deep Sequencing Facility of the MPI for Immunology and Epigenetics in Freiburg for access to their Illumina HiSeq2500 platform.

## Conflict of interest

The authors declare that the research was conducted in the absence of any commercial or financial relationships that could be construed as a potential conflict of interest.

## Publisher’s note

All claims expressed in this article are solely those of the authors and do not necessarily represent those of their affiliated organizations, or those of the publisher, the editors and the reviewers. Any product that may be evaluated in this article, or claim that may be made by its manufacturer, is not guaranteed or endorsed by the publisher.

## Abbreviations

Adamts, a disintegrin and metalloproteinase with thrombospondin motifs; AML, acute myeloid leukemia; AvSSMD*, robust strictly standardized median difference; BGR, background; Clpp, caseinolytic mitochondrial matrix peptidase proteolytic subunit; Clpx, ATP-dependent Clp protease ATP-binding subunit clpX-like; CSC, cancer stem cell; Dox, doxycycline; DUB, deubiquitinase; Ctsf, cathepsin F; FGR, foreground; GEO, Gene Expression Omnibus; HCC, hepatocellular carcinoma; Lonp1, Lon protease 1; MAD, median absolute deviation; miR-E, enhanced microRNA; Metap1/Metap2, methionine aminopeptidase 1/2; MMP, matrix metalloprotease; MPP, mitochondrial processing peptidase; NGS, next-generation sequencing; NOR, number of reads; Psma/Psmb/Psmc, proteasomal α/β/γ subunit; Psmd13, 26S proteasome non-ATPase subunit 13; pTCEBAC, TRE-Cyan-miR-E-PGK-BSDr-2A-Cherry; pTREBAV, TRE-dsRed-miR-E-PGK-BSDr-2A-Venus; RNAi, RNA interference; ROS, reactive oxygen species; SD, standard deviation; shRNA, short hairpin RNA; STRING, Search Tool for the Retrieval of Interacting Genes/Proteins; TRE, tetracycline response element; rtTA3, reverse tetracycline transactivator 3; Tysnd1, peroxisomal matrix protein trypsin domain-containing 1; UPS, ubiquitin-proteasome system; Usp46/7, ubiquitin-carboxyl terminal hydrolase 46/7; Uspl1, ubiquitin-specific peptidase like 1.
